# Changes in physical fitness and life skills among undergraduate students participating in a university physical education course

**DOI:** 10.3389/fspor.2026.1893216

**Published:** 2026-07-13

**Authors:** Kazuki Kaneda, Miyako Takano, Tomohiro Iwata, Ryudai Kaneko, Hiromi Shinno

**Affiliations:** Department of Health and Sports Sciences, Faculty of Health and Medical Sciences, Kyoto University of Advanced Science, Kyoto, Japan

**Keywords:** health promotion, physical education, physical fitness, sports life skills, university students

## Abstract

**Background:**

University students often experience a decline in physical activity after enrolling in higher education, which may negatively affect their physical health and psychosocial development. University physical education courses may provide important opportunities to promote physical activity and support student development.

**Purpose:**

This study aimed to examine changes in physical fitness and life skills among undergraduate students participating in a university physical education course.

**Methods:**

A single-group pre-post study design was employed. Undergraduate students enrolled in a mandatory university physical education course participated in the study. Physical fitness was assessed using measures of flexibility, mobility, and trunk endurance. Life skills were evaluated using the Daily Life Skills Scale for College Students. Pre-post changes were analyzed using paired *t*-tests, and effect sizes were calculated using Cohen's *d*. To examine whether observed post-course differences remained after accounting for baseline values and academic year, ANCOVA was performed with post-course scores as the dependent variable, pre-course scores as covariates, and academic year as a fixed factor.

**Results:**

Significant improvements were observed in all physical fitness outcomes, including flexibility, mobility, and trunk endurance (all *p* < 0.01). Personal, interpersonal, and total life skills scores increased significantly after the course (all *p* < 0.05). However, exploratory analyses revealed no significant associations between changes in physical fitness and life skills. Distribution analyses indicated that the proportion of students classified into higher performance categories increased across all physical fitness measures.

**Conclusion:**

Participation in a university physical education course was associated with positive changes in physical fitness and life skills. The study findings highlight the potential educational value of a university physical education course in supporting the physical and psychosocial development of university students. However, the findings should be interpreted with caution given the single-group pre-post design.

## 1 Introduction

In recent years, a global decline in physical activity levels among university students has been widely reported. Transitioning to university life often involves changes in lifestyle, academic demands, and daily routines, which may contribute to reduced physical activity. Insufficient physical activity during this stage of life is considered problematic because behavioral patterns established during young adulthood may influence long-term health outcomes (1). Therefore, promoting physical activity among university students has become an important public health and educational priority (2). In support of this perspective, a recent systematic review and meta-analysis reported that physical activity interventions can positively influence mental health outcomes among undergraduate students (3).

University physical education (PE) courses provide an important opportunity to encourage physical activity and support student health. Participation in structured PE programs improves the physical fitness and functional movement capacity of university students (4–6). More recently, Pugliese et al. (7) demonstrated that a 24-week combined circuit training and mobility program implemented within an academic community was associated with improvements in physical fitness outcomes and body composition. Specifically, components such as flexibility, mobility, and muscular endurance represent the fundamental elements of human movement and serve as the basis for daily physical activity and exercise participation. Improvements in these physical functions may facilitate continued engagement in physical activity and contribute to the maintenance of overall health. This perspective is further supported by a recent systematic review and meta-analysis showing that physical activity interventions implemented in university settings are generally associated with favorable effects on physical activity participation and health-related outcomes (8). Therefore, university PE courses may represent a valuable opportunity to support the physical well-being of students.

In addition to the physical health benefits, participation in sports and physical activity may contribute to psychosocial development. Such psychosocial competencies are often referred to as life skills, defined as abilities that enable individuals to effectively deal with the demands and challenges of everyday life (9). In the context of sports and physical activity, life skills are described as psychosocial abilities that may be developed through sports experiences and potentially transferred to other domains of life. Various assessment tools have been developed to evaluate life skills among athletes and university students, including the Daily Life Skills Scale for College Students developed by Shimamoto et al. (10). Participation in sports and physical activity may contribute to the development of life skills such as goal setting, communication, and self-regulation (11, 12). These skills are considered valuable not only in the context of sports but also in academic, professional, and social environments.

From this perspective, PE and sports activities are increasingly being viewed as educational settings that may support both physical and psychosocial development, and indeed, research has shown that participation in PE and sports can positively influence psychological and social outcomes such as self-efficacy, social competence, and well-being (13, 14). Because physical activity levels often decline after students enter university, PE courses may serve as a critical context in which both physical health and psychosocial competencies can be promoted. A recent systematic review also highlighted the potential of sport and physical education settings to foster positive youth development and psychosocial competencies (15). Despite these potential benefits, relatively few studies have simultaneously examined changes in physical fitness and life skills in the context of university PE. Most previous research has focused on improvements in physical fitness or the relationship between participation in sports and psychosocial development. Consequently, limited evidence exists regarding how university PE courses simultaneously contribute to both physical and psychosocial development. Moreover, many studies have evaluated changes in physical fitness only in terms of mean values, and relatively little attention has been paid to how the distribution of physical fitness levels may shift within the student population following participation in PE programs.

Because university PE courses provide educational opportunities for a large proportion of students, understanding how these courses influence both physical fitness and psychosocial development is important for evaluating their educational value. Clarifying these effects may contribute to a better understanding of the role of university PE in supporting the holistic development of students. Therefore, this study aimed to examine the effects of participation in a university physical education course on the physical fitness and life skills of undergraduate students. Specifically, this study evaluated changes in flexibility, mobility, and trunk endurance as indicators of physical fitness as well as changes in life skills, including personal and interpersonal skills. Additionally, exploratory analyses were conducted to examine whether changes in physical fitness were associated with changes in life skills.

## 2 Methods

### 2.1 Study design and participants

This study employed a pre-post quasi-experimental design to examine the changes in physical fitness and life skills following participation in a university PE course. The participants were undergraduate students enrolled in a mandatory university PE course titled “Sport Life Skills” at a university in Japan. In total, 221 students enrolled in the course and completed at least one assessment. Thirty-six students completed at least one assessment but did not complete any pre-post assessment pair and were therefore not included in the subgroup analyses. No *a priori* sample size calculation was conducted because all students enrolled in the course were invited to participate, and the analyses were based on available course data. Among them, 141 completed both pre- and post-course physical fitness assessments and were included in the physical fitness analysis. Eighty-four students completed both the pre- and post-course life skills questionnaires and were included in the life skills analysis. A subset of 40 students completed both the fitness and life skills assessments and were included in the exploratory analyses examining the associations between changes in physical fitness and life skills. The course was conducted over an academic semester and comprised structured fitness training sessions designed to improve flexibility, mobility, muscular endurance, balance, agility, and muscle strength.

### 2.2 PE course

The course consisted of 15 sessions conducted over an academic semester. The course was delivered across 10 classes taught by four instructors, with each class having 10–30 students. Before the beginning of the semester, instructors coordinated the course structure and ensured consistency of instructional content across classes. Each session lasted 90 min and followed a standardized structure. Students first spent approximately 5 min setting goals related to the session theme and recording them in a course reflection notebook, followed by a brief warm-up period. The main part of the session consisted of two structured exercise components (approximately 30 min each), the session concluded with a reflection period during which students reviewed their goal attainment and participation and documented their reflections in the course reflection notebook. The first exercise component was designed to prepare students for the second, more focused training component. For example, during flexibility-focused sessions, students first performed self-myofascial release exercises using foam rollers, followed by stretching exercises. Representative examples of exercise activities used throughout the course are presented in Supplementary Table S1. In addition to flexibility-focused activities, mobility-focused sessions incorporated yoga-based movements, range-of-motion exercises, and play-based activities designed to encourage whole-body movement. Trunk-endurance sessions included exercises aimed at improving core stability, such as gluteal activation exercises and plank-based training. Exercises were generally performed for multiple sets or repeated trials, and the overall difficulty and exercise volume gradually increased throughout the semester according to the session objectives. Recreational physical activities, including throwing games, running activities, walking, and jump-rope exercises, were also incorporated throughout the semester to promote overall physical fitness and movement competence. Although the overall session structure and learning objectives were standardized across instructors, specific exercise selection was adapted according to the session theme and class characteristics. The course was designed to improve multiple components of physical fitness, including flexibility, mobility, muscular endurance, balance, agility, and muscular strength. During this semester, particular emphasis was placed on flexibility, mobility, and physical fitness. Each session was organized around a specific theme, and students were encouraged to establish goals and reflect on their progress throughout the semester. Physical fitness assessments were conducted during Sessions 5 (baseline) and 14 (post-intervention). In addition to the regular notebook-based reflections conducted during each session, students completed structured reflective assignments during Sessions 5 and 14 to promote self-monitoring and goal setting. During Session 5, students reviewed their previous exercise habits, interpreted their assessment results, and established specific goals and action plans related to flexibility, mobility, and trunk endurance. During Session 14, students compared their pre- and post-assessment results and reflected on factors that may have contributed to their outcomes. These activities were intended to encourage self-reflection on physical activity behaviors and increase awareness of physical development throughout the semester.

### 2.3 Physical fitness assessment

Physical fitness was assessed by field-based measurements of flexibility, mobility, and trunk endurance. These components were selected because they represent fundamental elements of physical function related to joint range of motion, functional movement capacity, and core stability, which are important for injury prevention and overall physical performance in physically active populations. In addition, these assessments can be easily implemented in educational settings and are widely used in PE and sports science research. Flexibility was assessed using the standing trunk flexion test (standing forward bend) by using a standardized measurement device (Tanno Manufacturing Co., Japan). This test is conceptually similar to the widely used sit-and-reach test, which is commonly employed to evaluate the flexibility of the lower back and hamstrings (16). The participants performed two trials following the manufacturer's instructions, and the higher measurement value was recorded. The measurements were verified by the course instructors to ensure accuracy. Mobility was evaluated using a digital long-sitting forward-reach test (Takei Scientific Instruments Co., Japan). The participants performed two trials according to standardized procedures, and the highest measurement value was used for the analysis. Trunk endurance was assessed using a plank-hold test. The participants maintained the plank-forearm position on a mat supported by their forearms and toes. The time was recorded from the starting signal until any part of the body touched the floor or the posture could no longer be maintained, with the maximum duration being 5 min. A 5-min upper limit was established to minimize participant burden and to ensure the feasibility of testing within a large-group educational setting. Participants who maintained the plank position for the full 5 min were assigned a score of 300 s for analysis. The plank test is widely used as a practical indicator of core endurance and trunk stability (17, 18). The test was performed once, and the duration was recorded in seconds. Mobility and trunk endurance measurements were performed in pairs, with one student performing the test and the other recording the duration or value. To ensure standardized testing procedures, course instructors supervised all assessments.

### 2.4 Life skills assessment

Life skills were assessed using the Daily Life Skills Scale for College Students, developed by Shimamoto and Ishii (10). This scale was developed to evaluate psychosocial competencies required for successful adaptation to university life and has demonstrated acceptable reliability and validity among university students. The scale consists of 24 items across eight subdomains: planning, knowledge summarization, self-esteem, positive thinking, affinity, leadership, sensitivity, and interpersonal manners. Participants responded to each item using a 4-point Likert scale ranging from 1 (“not at all applicable”) to 4 (“very applicable”), with higher scores indicating greater life skills acquisition. Following the framework proposed by Shimamoto and Ishii (10), the eight subdomains were aggregated into personal life skills (planning, knowledge summarization, self-esteem, and positive thinking) and interpersonal life skills (affinity, leadership, sensitivity, and interpersonal manners). Each domain consisted of four subscales and 12 items. Total life skills scores were calculated as the sum of the personal and interpersonal life skills scores. The questionnaire was administered at the beginning and end of the semester. The Daily Life Skills Scale for College Students has previously demonstrated acceptable internal consistency, with Cronbach's *α* coefficients of 0.74 for personal life skills and 0.75 for interpersonal life skills in the original validation study (10).

### 2.5 Statistical analysis

**Figure 1:**
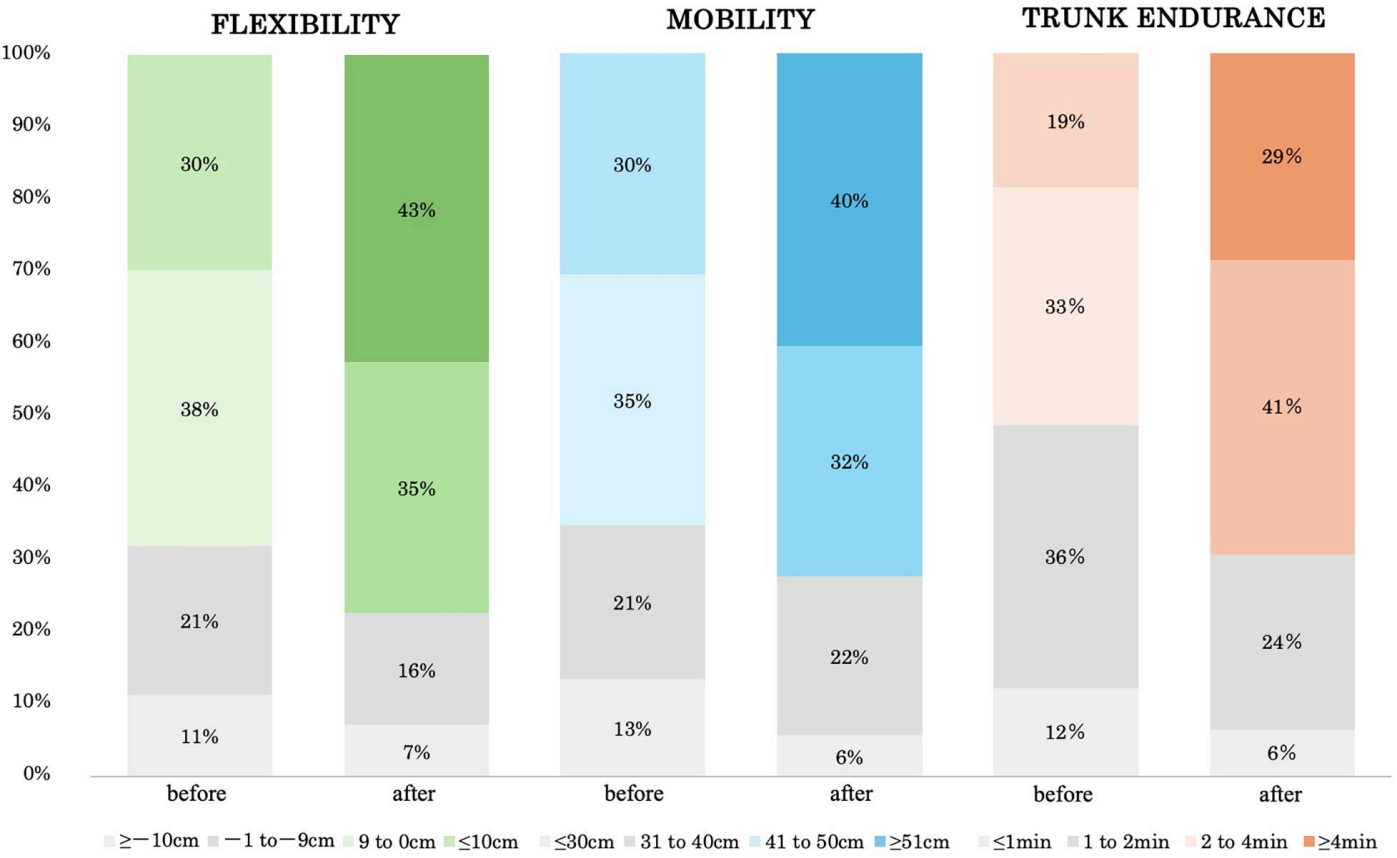
Distributional of physical fitness performance categories before and after the course. Flexibility categories were based on sit-and-reach performance (≤−10 cm, −9.9–0 cm, 0.1–9.9 cm, and ≥10 cm). Mobility categories were based on reach distance during the mobility assessment (≤40 cm, 41–50 cm, and ≥51 cm). Trunk endurance categories were based on plank holding time (<1 min, 1–2 min, 2–3 min, 3–4 min, and ≥4 min).

All statistical analyses were performed using SPSS version 31 (IBM Corp., Armonk, NY, USA). Normality of data distribution was assessed using the Shapiro–Wilk test. Baseline characteristics were compared according to assessment completion status to evaluate potential attrition-related differences among participants. Paired *t*-tests were used to examine the pre- and post-changes in physical fitness and life skills outcomes. Effect sizes (Cohen's *d*) were calculated by dividing the mean pre-post difference by the standard deviation of the difference scores. To examine whether post-course outcomes remained associated with academic year after accounting for baseline values, ANCOVA was performed with post-course scores as the dependent variable, pre-course scores as covariates, and academic year as a fixed factor. For descriptive purposes, the distribution of participants across performance categories was examined before and after the course. The performance categories shown in Figure 1 were created for descriptive purposes and were used solely to facilitate visualization of distributional changes over time. Exploratory Pearson correlation analyses were conducted among participants who completed both physical fitness and life skills assessments (*n* = 40) to examine the association between changes in physical fitness and life skills. Statistical significance was set at *p* < 0.05.

### 2.6 Ethics statement

This study was approved by the institutional ethics review board of the authors' university (approval number: 25M21). All participants provided informed consent before participation.

## 3 Results

Baseline characteristics according to assessment completion status are presented in Table 1. Age differed significantly among the fitness-only, LS-only, and combined assessment groups (*p* = 0.043), whereas no significant differences were observed in height or body weight. The sex distribution did not differ significantly between the groups (*p* = 0.106). The academic year distribution showed a significant difference between the groups (*p* < 0.001).

**Table 1:** Baseline characteristics by assessment completion status.

Variable	All *n* = 221	Fitness only group *n* = 101	LS only group *n* = 44	Combined assessment group *n* = 40	*F*-value	*p*-value
Age (years)	18.9 ± 1.1	18.7 ± 1.2	19.1 ± 0.6	19.0 ± 1.1	2.811	0.043
Height (cm)	165.2 ± 8.9	165.2 ± 8.7	164.7 ± 10.6	165.9 ± 7.6	0.093	0.964
Weight (kg)	60.3 ± 14.5	60.9 ± 13.5	58.5 ± 17.3	58.9 ± 13.1	0.374	0.722
Baseline flexibility (cm)	4.9 ± 10.4	5.5 ± 10.8	—	5.4 ± 10.0		
Baseline mobility (cm)	43.4 ± 10.6	43.7 ± 10.9	—	42.4 ± 9.9		
Baseline trunk endurance (s)	143.1 ± 82.8	150.9 ± 85.1	—	123.2 ± 73.1		
Sex, *n* (%)						0.106
Male	111 (50.2%)	44 (43.6%)	25 (56.8%)	27 (67.5%)		
Female	107 (48.4%)	55 (54.5%)	18 (40.9%)	13 (32.5%)		
Other	3 (1.4%)	2 (2.0%)	1 (2.3%)	0 (0.0%)		
Academic year, *n* (%)						<0.001
First year	107 (48.4%)	69 (68.3%)	6 (13.6%)	19 (47.5%)		
Second year	105 (47.5%)	25 (24.8%)	38 (86.4%)	20 (50.0%)		
Fourth year	9 (4.1%)	7 (6.9%)	0 (0.0%)	1 (2.5%)		

Values are presented as mean ± standard deviation or number (%). Group comparisons were performed using one-way ANOVA for continuous variables and chi-square tests for categorical variables. The baseline physical fitness variables were not compared across all groups because the LS questionnaire-only group did not answer the physical testing questions. ANOVA, analysis of variance; LS, life skills.

“All” includes all students who completed at least one assessment during the course (*n* = 221). The Fitness only, LS only, and Combined assessment groups include students who completed the corresponding pre-post assessment pairs. The remaining 36 participants completed at least one assessment but did not complete any pre-post assessment pair and were therefore excluded from subgroup analyses.

Significant improvements were observed in all physical fitness outcomes after the course (Table 2). Flexibility, mobility, and trunk endurance significantly increased from the pre- to the post-assessment period (all *p* < 0.01), indicating improved physical performance with small-to-moderate effect sizes. Flexibility and mobility remained significantly associated with post-course improvements after adjustment for academic year. However, trunk endurance showed a significant academic year effect in the adjusted model (ANCOVA, *p* < 0.001).

**Table 2:** Changes in physical fitness outcomes before and after the university physical education course (*n* = 141).

Outcome	Before the course (Pre; mean ± SD)	After the course (Post; mean ± SD)	Mean change	*t*-value	*p*-value	Cohen's *d*	ANCOVA F (Year)	ANCOVA *p* (Year)
Flexibility (cm)	4.9 ± 10.4	7.2 ± 10.1	2.3	4.70	<0.001	0.396	0.857	0.429
Mobility (cm)	43.6 ± 10.7	46.9 ± 9.6	3.3	5.56	<0.001	0.468	1.235	0.294
Trunk endurance (s)	143.1 ± 83.1	171.0 ± 89.3	27.9	5.42	<0.001	0.456	10.87	<0.001

Values are presented as mean ± standard deviation. Pre-post comparisons were performed using paired *t*-tests. Effect sizes were calculated using Cohen's *d*. Additional analyses adjusted for academic year were performed using ANCOVA. ANCOVA, analysis of covariance.

Significant improvements were also observed in personal, interpersonal, and total life skills scores following the course (Table 3). Scores for all domains significantly increased from the pre- to the post-assessment period (all *p* < 0.05), with small effect sizes. These improvements remained significant after controlling for the academic year. Exploratory correlation analyses among participants who completed both assessments (*n* = 40) revealed no significant associations between changes in physical fitness outcomes and changes in personal, interpersonal, or total life skills scores (Table 4). Correlation coefficients were generally small, and all associations were non-significant (all *p* > 0.05).

**Table 3:** Changes in life skills outcomes before and after the university physical education course (*n* = 84).

Outcome	Before the course (Pre; mean ± SD)	After the course (Post; mean ± SD)	Mean change	*t*-value	*p*-value	Cohen's *d*	ANCOVA F (Year)	ANCOVA *p* (Year)
Personal skills	30.4 ± 6.5	32.9 ± 5.9	2.5	2.62	0.010	0.286	0.047	0.955
Interpersonal skills	33.9 ± 5.9	36.0 ± 5.5	2.1	2.49	0.015	0.271	0.733	0.483
Total LS	64.2 ± 11.2	68.9 ± 10.3	4.7	2.88	0.005	0.314	0.047	0.955

Values are presented as mean ± standard deviation. Pre-post comparisons were performed using paired *t*-tests. Effect sizes were calculated using Cohen's *d*. Additional analyses adjusted for academic year were performed using ANCOVA. ANCOVA, analysis of covariance.

**Table 4:** Correlations between changes in physical fitness outcomes and life skills scores (*n* = 40).

Physical fitness change	Personal Skills		Interpersonal Skills		Total LS	
	*r* (95% CI)	*p*	*r* (95% CI)	*p*	*r* (95% CI)	*p*
Flexibility	0.021 (−0.293, 0.330)	0.900	0.169 (−0.151, 0.456)	0.298	0.104 (−0.214, 0.403)	0.522
Mobility	−0.005 (−0.316, 0.307)	0.973	−0.049 (−0.355, 0.266)	0.762	−0.030 (−0.339, 0.284)	0.853
Trunk endurance	−0.041 (−0.348, 0.274)	0.799	0.069 (−0.248, 0.372)	0.672	0.013 (−0.300, 0.323)	0.937

Values are Pearson correlation coefficients (r) with 95% confidence intervals (CI). Statistical significance was set at *p* < 0.05.

The distributions of flexibility, mobility, and trunk endurance shifted toward higher performance categories after the course (Figure 1). For flexibility, the proportion of participants achieving ≥10 cm increased from 30% (42/141) to 43% (60/141), whereas the proportion in the lowest category (≤−10 cm) decreased from 11% (16/141) to 7% (10/141). Similar shifts toward higher performance categories were observed for mobility and trunk endurance. For trunk endurance, the proportion of participants maintaining the plank position for ≥4 min increased from 19% to 29%.

## 4 Discussion

This study examined within-group changes in physical fitness and life skills among undergraduate students participating in a university PE course. The results showed significant improvements in all physical fitness outcomes, including flexibility, mobility, and trunk endurance, after the course. Additionally, significant improvements were observed in scores for personal, interpersonal, and total life skills. However, no significant association was found between the changes in physical fitness and changes in life skills. These findings suggest that participation in university PE courses may contribute to physical as well as psychosocial development among university students. Because this study used a single-group pre-post design without a control group, these changes should be interpreted as within-group observations and not as evidence of a causal course effect.

Regarding physical fitness, significant improvements were observed in flexibility, mobility, and trunk endurance. Previous studies have reported that participation in PE classes and physical activity programs among university students can improve musculoskeletal fitness and functional movement capacity (2–4). Similarly, Maisto et al. (19) reported improvements in physical fitness outcomes following a structured physical activity program conducted within an academic environment, further supporting the potential value of organized exercise opportunities in educational settings. In addition, educational interventions designed to promote physical activity have been shown to influence exercise behavior among university students (6). The physical fitness components evaluated in the present study, flexibility, mobility, and trunk endurance, represent the fundamental elements of human movement and serve as the foundation for daily physical activity and exercise performance. Therefore, the present findings suggest that positive changes in basic physical fitness may occur during participation in university PE courses. In the adjusted analyses, academic year was significantly associated with post-course trunk endurance after accounting for baseline values. This finding suggests that the observed changes in trunk endurance may have been influenced partly by differences in academic year composition and should be interpreted more cautiously than the flexibility and mobility findings.

In addition to examining mean changes, this study evaluated changes in the distribution of performance categories for physical fitness outcomes. The results showed that an increased proportion of students were classified into higher performance categories across all indicators, including flexibility, mobility, and trunk endurance. For example, with regard to flexibility, the proportion of students achieving values of ≥10 cm increased, whereas the proportion of students in the lowest category decreased. Similar trends were observed for mobility and trunk endurance. These findings suggest that the PE course did not simply improve performance in a limited number of students, but might have elevated the overall physical fitness level of the student population. Furthermore, significant improvements in life skills were also observed. Life skills are defined as psychosocial competencies that enable individuals to deal effectively with the demands and challenges of everyday life (9). Previous research has indicated that participation in sports and physical activity may facilitate the development of life skills such as goal setting, communication, and self-regulation (11, 20). The present findings support the findings of these studies and suggest that university PE courses may contribute to the development of psychosocial competencies. This interpretation is consistent with recent systematic review evidence indicating that sport and physical education settings can facilitate positive youth development and the acquisition of psychosocial competencies through structured learning experiences (15). One possible explanation for the improvement in life skills may be related to the course's instructional design. Goal setting and self-monitoring are considered key components of self-regulated learning processes that facilitate behavioral and psychosocial development (21). In the present study, the PE course incorporated reflective assignments that encouraged students to set goals and monitor their physical activity. Students were asked to review their previous exercise habits; establish goals related to flexibility, mobility, and trunk endurance; and then evaluate their progress toward these goals. These reflective learning processes may promote self-regulated learning and contribute to the development of personal and interpersonal life skills.

Despite improvements in both physical fitness and life skills, no significant correlations were found between the changes in these outcomes. However, this finding should be interpreted with caution because the correlation analysis was exploratory and included only participants who completed both assessments (*n* = 40). In addition, correlations based on change scores may be statistically unstable and sensitive to measurement variability. Therefore, the absence of significant associations should not be interpreted as evidence that physical fitness and life skills development are unrelated. Nevertheless, the present findings suggest that improvements in physical fitness and psychosocial development were not clearly associated in this sample, although further research is needed to clarify their relationship.Improvements in physical fitness are likely influenced by physiological adaptations to exercise stimuli, whereas life skills development may be more strongly influenced by educational experiences such as social interaction, self-reflection, and goal setting within the course environment. Future studies with larger samples are needed to further examine the relationships between changes in physical fitness and life skills.

From an educational perspective, the findings of this study highlight the broader educational value of university PE. Participation in PE has been shown to influence not only physical health but also psychosocial outcomes, such as self-efficacy and social competence (13, 22). Because physical activity levels tend to decline after students enter university, PE courses may serve as important opportunities to promote physical activity. The present findings suggest that university PE courses may contribute not only to improvements in physical fitness but also to life skills development among university students.

This study has some limitations. First, it employed a pre-post design without a control group; therefore, causal relationships between participation in the PE course and the observed outcomes cannot be definitively established. Second, this study was conducted at a single university, which may limit the generalizability of the findings to other educational contexts. Third, life skills were assessed using a self-reported questionnaire, which may have been subject to response bias. In addition, the 5 min upper limit used for the plank test may have introduced a ceiling effect among participants with higher levels of trunk endurance. Finally, substantial attrition occurred across assessments, and participants who completed different components of the study differed in age and academic year. Although additional analyses adjusted for academic year, the possibility of attrition and selection bias cannot be excluded. Future studies should consider employing controlled study designs and including participants from multiple universities to further examine the impact of university PE courses on both physical fitness and life skills development.

In conclusion, positive changes in physical fitness and life skills were observed among students during participation in a university PE course. Although the single-group pre-post design precludes causal inference, these findings suggest that university PE courses may provide educational settings that support both physical and psychosocial development among university students.

## Data Availability

The raw data supporting the conclusions of this article will be made available by the authors, without undue reservation.
