# Supplementary material for: Changes in physical fitness and life skills among undergraduate students participating in a university physical education course
Source: Front Sports Act Living. 2026 Jul 13;8:1893216. doi: 10.3389/fspor.2026.1893216 (PMC13403327; doi:10.3389/fspor.2026.1893216)
Supplement: Supplementary file 1 [file Table1.docx]

Supplementary Table S1. Representative examples of exercise activities used in the university PE course.

| **Session theme** | **Representative activities** |
| --- | --- |
| Flexibility | Foam roller exercises, massage ball release, static stretching, dynamic stretching |
| Mobility | Yoga-based movements, mobility drills, play-based movement activities |
| Trunk endurance | Gluteal activation exercises, pelvic control exercises, plank variations |
| General fitness | Walking, running activities, jump-rope exercises, throwing games |

Note: Representative activities are provided as examples and do not constitute a complete list of all exercises performed during the course.
